# Longevity strategies in response to light in the reef coral *Stylophora pistillata*

**DOI:** 10.1038/s41598-020-76925-2

**Published:** 2020-11-17

**Authors:** Alexandre Ottaviani, Rita Eid, Didier Zoccola, Mélanie Pousse, Jean-Marc Dubal, Edwige Barajas, Karine Jamet, Kevin Lebrigand, Pascal Lapébie, Christian Baudoin, Marie-Josèphe Giraud-Panis, Alice Rouan, Gallic Beauchef, Christelle Guéré, Katell Vié, Pascal Barbry, Sylvie Tambutté, Eric Gilson, Denis Allemand

**Affiliations:** 1 Medical School of Nice, CNRS, INSERM, IRCAN, Université Côte d’Azur, Nice, France; 2 Centre Scientifique de Monaco, Monaco, Monaco; 3 Université Côte d’Azur, CNRS, IPMC, 06560 Sophia Antipolis, France; 4 Laboratoires Clarins, 12 avenue de la porte des Ternes, 75017 Paris, France; 5 Department of Genetics, CHU, Nice, France

**Keywords:** Marine biology, Cell biology, Circadian rhythms, Senescence

## Abstract

Aging is a multifactorial process that results in progressive loss of regenerative capacity and tissue function while simultaneously favoring the development of a large array of age-related diseases. Evidence suggests that the accumulation of senescent cells in tissue promotes both normal and pathological aging. Oxic stress is a key driver of cellular senescence. Because symbiotic long-lived reef corals experience daily hyperoxic and hypoxic transitions, we hypothesized that these long-lived animals have developed specific longevity strategies in response to light. We analyzed transcriptome variation in the reef coral *Stylophora pistillata* during the day–night cycle and revealed a signature of the FoxO longevity pathway. We confirmed this pathway by immunofluorescence using antibodies against coral FoxO to demonstrate its nuclear translocation. Through qPCR analysis of nycthemeral variations of candidate genes under different light regimens, we found that, among genes that were specifically up- or downregulated upon exposure to light, human orthologs of two “light-up” genes (HEY1 and LONF3) exhibited anti-senescence properties in primary human fibroblasts. Therefore, these genes are interesting candidates for counteracting skin aging. We propose a large screen for other light-up genes and an investigation of the biological response of reef corals to light (e.g., metabolic switching) to elucidate these processes and identify effective interventions for promoting healthy aging in humans.

## Introduction

Aging is classically defined as a progressive loss of organ and organismal function, renewal potential, and stress resistance. At the cellular level, aging represents the accumulation of unrepaired or irreparable damage leading to cellular senescence^1^. Although aging affects all organisms, its mechanisms have mainly been studied in a limited number of model organisms (yeasts, nematodes, mice, and *Drosophila*)^2,3^. Exploration of the aging process of non-model organisms is crucial for developing a more generalized understanding of aging. Organisms with unique habitats or life histories are particularly likely to have distinct biological processes useful in the development of novel environmental or medical applications.

In this context, cnidarians such as hydra and corals emerge as potent models of aging as their genetic individuals exhibit extreme lifespan, regeneration capacity, and resistance to ultraviolet (UV) light or oxic stress^4–7^. Reef-building corals host photosynthetic endosymbiotic dinoflagellate algae (family Symbiodinaceae), commonly called zooxanthellae, in their endodermal cells. As a result of the photosynthetic activity of these algae, coral cells undergo drastic and rapid nycthemeral variation in exposure to oxygen and/or reactive oxygen species (ROS); the extreme limits of this variation correspond to hypoxia (i.e., < 5% O_2_) at night and hyperoxia (i.e., > 60% O_2_) during the day^8–10^. However, at the same time these animals can live and expand clonally for decades in aquaria and in the field, and the ages of some wild genetic individuals have been estimated to be more than several hundreds of years^11,12^. As previously discussed^5^, we consider in this work that a coral individual refers to the syngenic colony as a whole and not to an isolated polyp. Thus, reef building corals represent a paradox in terms of current theories of aging, which characterize high ROS levels as an accelerant of aging^13^. Some specific mechanisms of oxic stress resistance have been identified in corals^6,7,14–16^; however, their contribution to the extreme longevity of these animals remains unknown. Obviously, as exposure to daylight is the main driver of symbiotic corals physiology, these resistance mechanisms are linked to circadian rhythms^6,17–19^, but, interestingly, even in mammals, circadian molecular clocks are crucial for stress management and their dysfunctions can result in aging phenotypes^20^. This suggest very ancient evolutionary links between these processes, echoing with the common origin of cryptochrome proteins from the core molecular clocks and light-induced UV damage-repairing photolyases^21^.

In this study, we investigated nycthemeral transcriptional variation in the coral *Stylophora pistillata* and the expression of genes involved in the nycthemeral oxic transition as well as those related to anti-senescence properties in humans. Our results help elucidate the biological response of corals to light and contribute to the identification of new genes and biological pathways involved in human aging.

## Results

### A transcriptomic signature of the nycthemeral oxic transition

**Figure 1:**
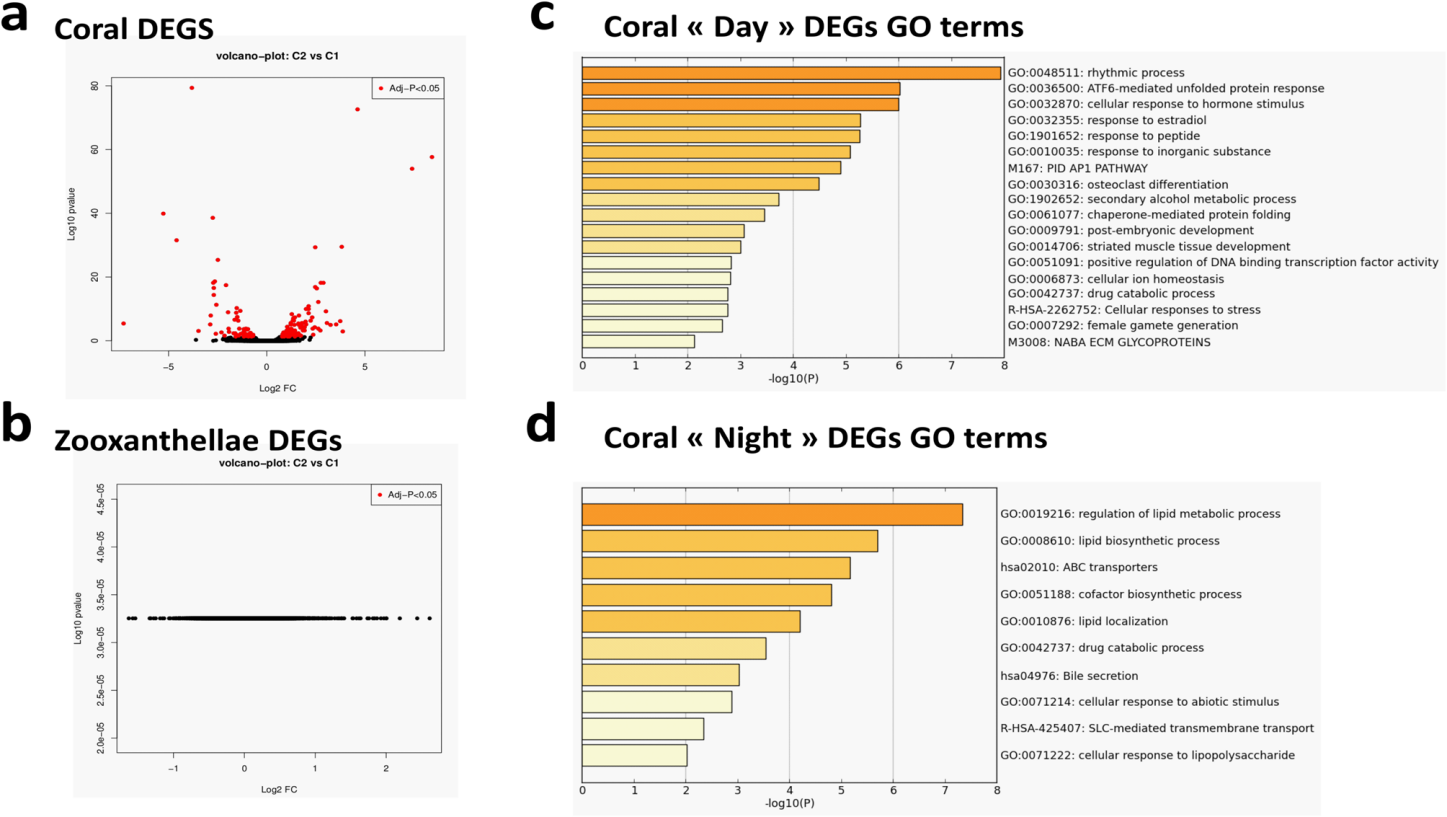
Day vs. night transcriptomic signatures. (**a**,**b**) Volcano plots of significantly differentially expressed genes (DEGs) in *Stylophora pistillata* (**a**) or *S. microadriaticum* (**b**) transcriptomes, generated with the R software package. (**c**,**d**) Bar charts representing significantly enriched terms identified in Metascape annotation analyses among significantly upregulated (**c**) or downregulated (**d**) genes in Day or Night transcriptomic signatures of *S*. *pistillata*. Color gradient is scaled to the represented −log10(P-value).

Isogenic samples of *Stylophora pistillata* were collected 1 h before the end of each 12 h:12 h photoperiod of light:dark conditions, and we will respectively refer to them as “day” and “night” samples. Total RNA was extracted and used to prepare cDNA libraries that were sequenced with SOLiD technology (Fig. S1). Reads were mapped against the *Stylophora pistillata* gene models^22^ to obtain relative expression data. Using an adjusted P-value cutoff of 0.05, we identified 151 genes that were significantly modulated between these two conditions (Fig. 1a, Table S3) and contained 122 nonredundant annotations. When reads were mapped against the *Symbiodinum microadriaticum* gene models^23^, no significantly up- or downregulated genes were obtained given our P-value threshold (Fig. 1b) although these mapped reads represented about a third (31.33%) of total mapped reads (Table S1). This inability to identify significantly modulated *Symbiodinium* genes from our RNA sequencing (RNA-seq) samples was surprising. The relatively low sequencing depth of our RNAseq study (Table S1) precludes the analysis of low expressing DEGs. However, this is unlikely to explain the absence of algal DEGs since large and similar numbers of reads were observed for key photosynthesis genes in both “day” and “night” conditions (Table S2). Moreover, similarly very limited transcriptional changes have been reported in previous studies in dinoflagellates for both axenic^24^ and coral-hosted^25^ cultures upon exposure to thermal or other stresses. Thus, it appears that dinoflagellates mainly regulate daily or short-term expression through posttranslational mechanisms that may consist of regulating small RNA^26^ or trans-splicing with spliced leader (SL) sequences^24^. Regulation involving these mechanisms might be complex^24^, and their effects may be invisible to polyadenylated mRNA-based RNA-seq differential expression analyses, such as the present study.

Gene ontology (GO) and pathway analyses were performed with 102 orthologous human genes among the 122 *S*. *pistillata* nonredundant differentially expressed genes (DEGs), determined by identifying the best BLAST hits against the UniProtKB/Swiss-Prot database. GO terms related to lipid metabolism and small-molecule transport were overrepresented among downregulated genes (i.e., more present in night samples; Fig. 1d), and those associated with circadian rhythms, cellular responses to stimuli, and stress and protein stability were overrepresented among upregulated genes (i.e., more present in day samples; Fig. 1c). These results are consistent with previous studies of cnidarian transcriptomics that have reported similarly enriched terms among DEGs between day and night samples in *Acropora cervicornis*^15^ and *A*. *millepora*^18^ and heat or light stress samples in *A*. *hyacinthus*^27^ and *S*. *pistillata*^28–30^. Noteworthy, we did not find significative enrichment for biological processes such as DNA repair and cell cycle^29^, although some of our DEGs had corresponding annotations: XPC and PHR involved in DNA repair while HSP68, CRY1, CALR, COT2, PHC1, ECT2, ATF4, ETS2, XPC; CB042, CLOCK and ANPRA are linked to cell cycle.

### FOXO is a master regulator of the nycthemeral oxic transition transcriptional signature

**Figure 2:**
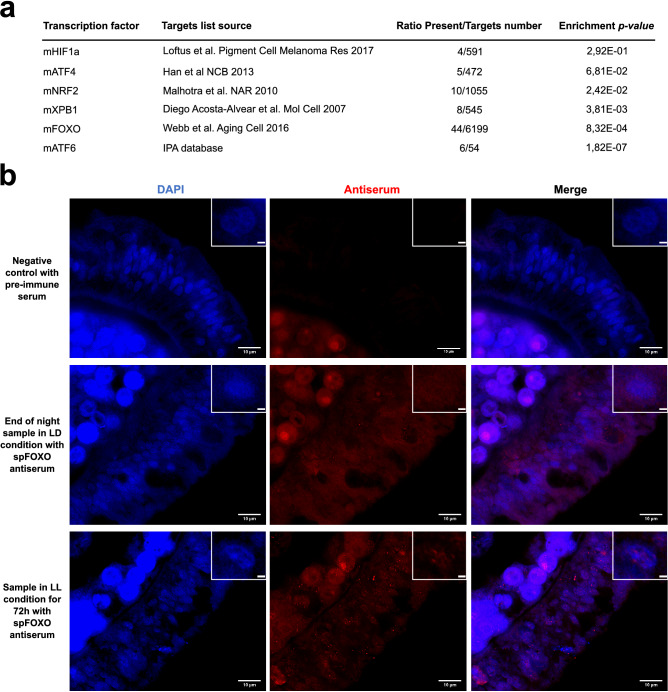
FOXO response in light-exposed coral. (**a**) Comparison of transcriptomic signatures with published lists of selected transcription factor targets^31–35^. (**b**) spFOXO activated in light-exposed coral tissue. Immunofluorescence labeling (red signal) with pre-immune serum (negative control) or spFOXO antiserum and DAPI counterstain (blue signal) of paraffin-embedded coral tissue from syngenic samples collected at night from a colony grown in light/dark conditions and at noon after 72 h of continuous light. An enlarged view of an isolated nucleus is provided in the top right corner, scale bar herein is 1 µm.

To determine the upstream factors regulating the transcriptomic signature of day and night alternation, we compared our signature to published lists of mouse genes known to be directly regulated by stress response master transcription factors XBP1, ATF4, and ATF6 for Unfold Protein Response (UPR), NRF2, and FOXO for oxidative stress and HIF1α for hypoxia (Table S4a)^31–35^. Published HIF1α and ATF4 target genes lists were not significantly overrepresented in the *S*. *pistillata* transcriptomic signature, whereas all other lists were (Fig. 2a), further suggesting endoplasmic reticulum and oxidative stresses pathways are involved. We found that most DEGs were FOXO targets in one or several of four different animal species for which FOXO chromatin immunoprecipitation DNA sequencing (ChIP-Seq) data are available (Figure S4, Table S4b)^35^, which suggests that FOXO is a master regulator of a large proportion of transcriptional responses to coral nycthemeral oxic transitions.

In classical models, FOXO activity is regulated by reversible phosphorylation and subcellular localization; its activation triggers its translocation into the nucleus. To investigate the regulation of putative nuclear translocation of spFOXO, we raised antibodies specifically directed against the spFOXO protein (Figure S5). In immunofluorescence experiments, we observed a clear punctuated FOXO pattern in the nuclei of *S*. *pistillata* tissue subjected to 72 h of continuous light compared to a control sample at the end of the scotophase that exhibited only diffuse cytoplasmic staining with spFOXO antibodies (Fig. 2b). Thus, spFOXO is activated in coral tissue on exposure to light, in agreement with the FOXO transcriptomic signature observed during the nycthemeral oxic transition.

### Light-regulated coral genes

Among all significantly modulated genes (Table 1), we selected 23 genes of interest that were representative of the GO terms and pathways. We analyzed their expression by quantitative reverse-transcription polymerase chain reaction (qRT-PCR) in new coral samples collected at the same nycthemeron times as those used in the RNAseq analysis. Among them, 11 genes were validated since they were consistently modulated in the same direction as observed in RNA-seq analyses and with |fold-change|> 2.

**Table 1:** Validation of selected genes of interest.

Gene symbol	qPCR	RNA seq
	LOG2 (FC)	LOG2 (FC)
ACOD	− 0.81	− 1.48
ACOX1	1.10	− 2.13
APLP	0.61	1.16
**CAH2**	**2.21**	**1.53**
**CATA**	**2.66**	**1.55**
**CLOCK**	**2.69**	**1.43**
**CRY1**	**1.63**	**2.48**
CRYD	− 1.19	− 0.90
ECT2	− 0.79	− 1.43
**EGR1**	**1.55**	**2.65**
**FOS**	**2.16**	**2.58**
HECD3	0.21	− 1.08
**HEY**	**4.13**	**3.82**
**LONF3**	**4.02**	**4.59**
MDR1	− 0.69	− 1.05
**MMP19**	**2.16**	**1.48**
MRC1	0.82	− 1.39
PAX3	0.89	2.72
**PHR**	**− 1.98**	**− 2.55**
**PTHD3**	**− 2.27**	**− 2.46**
SC5A9	2.52	− 1.28
SIK1	0.44	2.07
XPC	− 0.91	− 2.31

We selected 23 genes of interest among differentially expressed genes (DEGs) based on main pathways, ontology, and functional terms overrepresented in the transcriptomic data as tested by quantitative reverse-transcription polymerase chain reaction (qRT-PCR) using two reference genes (36B4 and β-actin) and compared them to RNA sequencing results in six independent samples collected at the exact same moments of the nycthemeron. We validated 11 genes with co-directional expression and │fold-change│ > 2 (bold) as candidate genes.

**Figure 3:**
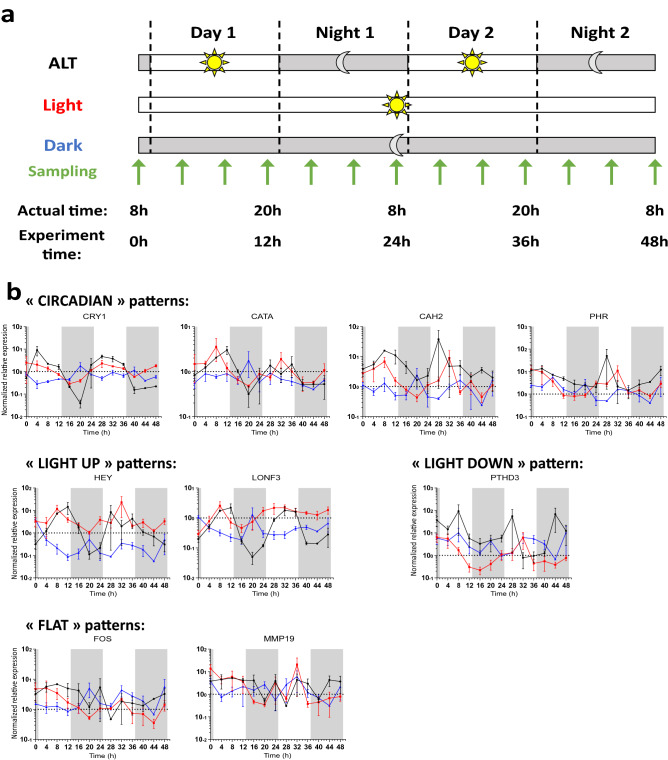
Circadian expression patterns of candidate genes. (**a**) Three samples were collected every 4 h during a 48 h period from isogenic *S*. *pistillata* colonies under conditions of alternating light and darkness (ALT), continuous light (Light), or continuous darkness (Dark). (**b**) Expression kinetics of candidate genes measured using RT-qPCR and ordered by the different patterns observed. White and grey backgrounds correspond to the actual/subjective photophase and scotophase, respectively.

We investigated whether these genes were regulated by a biological clock or directly by light. We performed an experiment in which isogenic coral microcolonies were collected in triplicate every 4 h during a 48 h period under three different conditions: a control condition similar to aquarium culture with daily alternation of 12 h of light and 12 h of darkness (ALT), continuous light (Light), and continuous darkness (Dark; Fig. 3a), and were then analyzed by qRT-PCR.

Using these results, we characterized different expression patterns (Fig. 3b). The expected oscillations were obtained in the ALT condition regarding the expression of the clock gene CRY1. Interestingly, continuous light preserved CRY1 oscillations despite a decrease in their amplitude. However, complete darkness resulted in a rather flat expression pattern, which suggests that oscillating CRY1 expression over a 24 h period may depend on the presence of light but not its alternation with darkness. We identified this pattern, also followed by catalase (CATA), carbonic anhydrase 2 (CAH2) and photolyase (PHR), as “circadian”.

Then we characterized other patterns, including a “light-up” pattern that was observed for the HEY gene; this pattern exhibited oscillations under ALT conditions but general gene expression upregulation under continuous light (Fig. 3). The same pattern was observed for the LONF3 gene.

By contrast, the “light-down” pattern exhibited by PTHD3 showed general gene expression downregulation under continuous light, with some conservation of oscillations, which suggests modulation by both light and the biological clock (Fig. 3).

Some other genes showed a relatively “flat” pattern, with no characteristic oscillations or recurrent changes and no clear effects of continuous light or darkness.

### Two light-up genes protect against senescence in human fibroblasts

**Figure 4:**
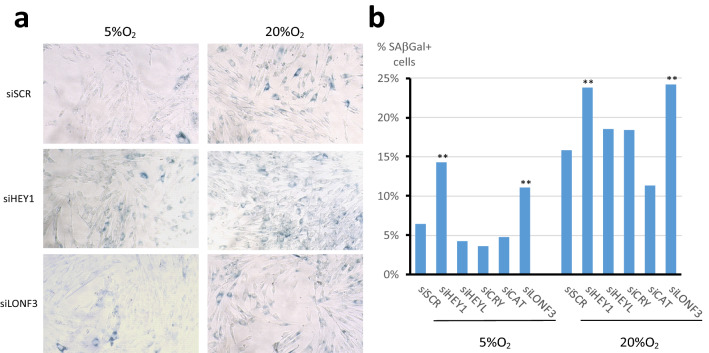
Knockdown of candidate gene orthologs in MRC5 fibroblasts. Non-senescent MRC5 cells were cultured in either 5% or 20% O_2_ and then transfected with indicated siRNA 96 h prior to senescence-associated β-galactosidase staining. (**a**) Representative photographs of labeled cells. (**b**) Results are expressed as percentages of labeled cells. A minimum of 200 cells were counted in each condition. P values were calculated using the binomial law, relative to the control condition, and corrected for multiple comparisons with the Bonferroni correction (α′ = 0.008333; **P-value < 10^–5^).

Because genes with circadian or light-dependent patterns participate in oxic transition protection mechanisms, we knocked down expression of their human orthologs in MRC5 fibroblasts cultured at 5% or 20% O_2_ and measured the proportion of cells displaying senescence-associated β-galactosidase (β-gal) staining (Fig. 4). We tested genes orthologous to the light-up genes including all three spHEY human orthologs (HEY1, HEY2, and HEYL). PHR has no human ortholog, and the KD of PTHD3 produced unsatisfactory results; therefore, these genes are not discussed further here. Although catalase KD had no significant impact, KD of genes orthologous to the light-up genes HEY and LONF3 increased the proportion of cells with β-gal staining at 5% and 20% O_2_, which indicates that these genes have longevity properties in human cells.

## Discussion

Two main conclusions can be drawn from the results of this study: FOXO is a master regulator of the nycthemeral oxic transition transcriptional signature in coral, and genes modulated by light in coral (light-up/-down genes) are novel candidates for stress resistance or longevity properties that may be conserved in orthologous species.

The FOXO family of transcription factors has been extensively associated with biological rhythms, stress responses, stem cells, and longevity in animals^36^. In cnidarians, FOXO plays a key role in determining *Hydra* self-renewal capabilities and apparent immortality by maintaining stemness in all three stem cell lineages of these organisms^37^. In the present study, FOXO was activated in corals upon stimulation with light, which suggests the existence of FOXO functions related to defense mechanism management in corals as previously hypothesized^38^. Stem cell compartments have not yet been characterized in *S*. *pistillata*; however, management of daily hyperoxia in progenitors is likely key in its exceptional resistance and longevity, and our results further suggest a suspected role of FOXO in the underlying cellular processes.

Investigation of the nycthemeral oxic transition mechanisms in corals is difficult as they may be mitigated by effects of the endogenous circadian clock, light stimulation independent of photosynthesis, and light stimulation dependent on photosynthesis (symbiotic relationships). Our RNA-Seq experimental design did not permit us to disambiguate the effects of these parameters because we focused on exploring global responses. Among our candidate genes, we identified two clock genes, CRY1 and Clock. However, our time-course analyses of candidate genes allowed us to refine interpretations of the contribution of the biological clock to regulating the expression of these genes.

Only a few studies have examined cnidarian models under conditions of continuous light^17,39^ or continuous darkness^18,21,40^, and none have explored both within the same experiment. Our results suggest that light (or its consequences, i.e. increased O_2_ concentration) is not directly linked to CRY1 gene expression amplitude but rather is necessary for the pattern of its oscillation over a 24 h period independent of a darkness phase. This finding suggests that light is a necessary kick-starter for endogenous clock oscillations and that oscillating expression of CRY genes is hardwired into their molecular pathways, which makes this molecular clock an intermediate between simple light-induced expression and an autonomously oscillating clock. Additional evidence, notably a more extended list of clock genes, will be required to prove this hypothesis and determine whether it represents a widespread strategy among cnidarians. As recent studies continue to reveal connections between the transcriptional clock, autonomously oscillating redox and metabolic systems, or aging, endosymbiotic cnidarians may prove to be interesting models for deciphering these relationships^19,20^.

Among our candidate genes that met all selection criteria, some might have been expected based on their known functions. Examples include CRY1, CATA, PHR, and CAH2. Catalase is well known for its antioxidant defense functions and has been extensively studied in cnidarians^41^. However, the pattern of its expression is surprisingly similar to that of CRY1 and is unaltered by continuous light treatment, which suggests that circadian control is the main regulator of this gene. Photolyase (PHR) has a blue light–induced UV-damaged DNA repair function that seems particularly relevant in endosymbiotic cnidarians but has been poorly investigated in these organisms^42^. In our study, PHR expression patterns did not account for clear clock- or light-induced modulations, although its expression seems to increase around subjective dawn in ALT or continuous light conditions. Carbonic anhydrase 2 (CAH2) activity provides inorganic carbon for both photosynthesis and calcification^43,44^; thus, circadian control of its expression is consistent because it is expected to fuel both processes in the context of light-enhanced calcification^45,46^.

It is interesting that human orthologs of the two candidate genes showing light-up modulation (LONF3 and HEY1) have anti-senescence properties in human fibroblasts. LONF3 is related to LON proteases, which are conserved across all kingdoms and implicated in proteostasis, a critical process for the turnover of damaged proteins, the failure of which is a hallmark of aging. LON proteases are also involved in mitochondrial UPR^47^. LONF3 shares its N-terminal substrate binding domain but has two zinc finger motifs. However, its specific function has not been reported in any model organism, except for a single screen in which its KD increased gH2AX phosphorylation in HeLa cells^48^, which suggests that it may contribute to protecting against DNA damage. HEY1 belongs to a family of bHLH transcription factors related to the *Drosophila* Hairy and Enhancer of Split genes. Hey genes are targets of the Notch and TGFb signalization pathways and repress other TFs as dimers recruiting HDACs^49^. Consistent with our data, the closely related HELT in *Nematostella vectensis* exhibits light-dependent oscillating expression^40^. A HEY family of TFs was recently associated with the maintenance of stem cell functional pools through p53 or lamin regulation in mice and *Drosophila*^50,51^ and with cancer cell self-renewal and differentiation in humans, which suggests that it may contribute to cell renewal homeostasis in physiological and aging contexts. PTHD3 belongs to the Patched Domain-Containing gene family, which is ancient in animal and plant evolution^52^. These genes are related to hedgehog signaling, sterol metabolism, and cell patterning; however, their precise functions remain elusive.

Overall, our candidate genes involved in metabolism and antioxidant defenses appear to rely mainly on circadian patterns, which suggests anticipation strategies. Transcription factors are more directly regulated by light, which suggests a role centered on defense adjustment to actual environmental conditions. This hypothesis is supported by our KD experiments in human fibroblasts, which revealed a protective role in their orthologs. Mechanisms behind both anticipation and adjustment strategies may be relevant to the aging process since molecular damages accumulate faster during time shifts between raises in damaging sources and corresponding defense mechanisms^1,19,20^. The precise functions and mechanisms of action of these candidate genes or their paralogs remain poorly described, which suggests that they may harbor original functions that are key to the exceptional resistance and longevity exhibited by corals and that they may have unsuspected benefits in humans.

In this study, we used an unconventional animal model with extreme properties to reveal unsuspected functions of orthologous systems. Although further study is required to fully understand their precise mechanisms and how evolution has altered their regulation, we identified candidate genes that likely contribute to stress resistance and longevity in corals and mammals. We also established a novel set of tools and techniques for this new coral model. Overall, our approach supports the idea that biodiversity can potentially help discover new mechanisms for human systems.

## Methods

Detailed methods for coral total RNA extraction, cDNA synthesis, transcriptomic data processing and spFOXO purification and antibody development are available in Supplementary data.

### Coral culture

Experiments were conducted with the tropical symbiotic scleractinian species *Stylophora pistillata* which is kept in long-term culture at the Centre Scientifique de Monaco. Colonies were supplied with water from the Mediterranean Sea (exchange rate 2% per hour) under controlled conditions, including a semi-open circuit, a temperature of 25 °C, salinity of 38, irradiance of 175 µmol photons m^−2^ s^−1^ on a 12:12 photoperiod. Corals were fed daily with frozen rotifers and twice a week with *artemia nauplii*. Microcolonies were propagated by cutting terminal portions of branches (6–10 mm in length) from three syngenic parent colonies, hung by nylon thread with a specific label in a dedicated aquarium under similar conditions, and left for 10 days for recovery prior to experiments. Day coral samples for RNA-seq were harvested and flash-frozen 1 h before the end of the light period; night samples were harvested 12 h later in the same manner. For circadian expression experiments, isogenic coral microcolonies were collected in triplicate and flashfrozen in liquid nitrogen every 4 h during a 48 h period under three different conditions: a control condition similar to aquarium culture with daily alternation of 12 h of light and 12 h of darkness (ALT), continuous light (Light), and continuous darkness (Dark). For conditions involving continuous light or darkness, coral colony fragments were placed in these conditions 24 h before the first samples were collected.

### Library preparation and sequencing

The concentration and quality of total RNA were assessed with a Bioanalyzer 2100 and RNA 6000 nano kit (5067–1511; Agilent). All total RNA samples with a RIN superior to 8 (Figure S2) were used for mRNA preparation using the MicroPoly(A)Purist kit (AM1919; Applied biosystems). mRNA quality was assayed using 2100 Bioanalyzer and the RNA 6000 pico kit (Figure S3). mRNA with a good profile on Bioanalyzer were used for libraries preparation. mRNA was prepared with the MicroPoly(A) purist kit (AM1919; Applied Biosystems). We used 1 µg mRNA per sample to construct libraries using the SOLiD Total RNA-Seq Kit (4445374; Thermo Fisher) and they were barcoded with SOLiD RNA barcoding kit (4427046; Applied Biosystems). Library concentrations were measured with quantitative PCR with the Kapa DNA quantification kit (KK4806; Kapa). Library quality was measured with a Bioanalyzer 2100 and high-sensitivity DNA kit (5067–4626; Agilent). Fragment sequencing was achieved through emulsion PCR, bead deposition, and ligation-based sequencing performed with a SOLiD 3 sequencer and the Top Paired end sequencing kit (4459182; Applied Biosystems) according to the manufacturer’s instructions. 3 samples were sequenced for each condition and each sequencing generated 30 to 55 million reads.

### qRT-PCR

To ensure useful comparison of all quantitative PCR results and setup conditions for all primer pairs, we generated an artificial reference sample using equal quantities of cDNA from all 109 quality control–validated samples from the 48 h expression kinetics experiment. We diluted this reference sample 5, 10, 20, 40, 80, 320 and 1280 times to establish standard curves for primer pair parameters determination and the 40 times dilution was prepared in larger quantities and used as a common normalizer sample for all experiments (D40Ref).

All primer pairs were designed in silico on exon junctions whenever possible with Primer3 (http://bioinfo.ut.ee/primer3/) and checked for specificity with BLAST searches (https://blast.ncbi.nlm.nih.gov/Blast.cgi) and for amplicon secondary structures with mfold (http://mfold.rna.albany.edu/?q=mfold/DNA-Folding-Form). Primer pairs were ordered from Sigma and tested for specificity by endpoint PCR using D40Ref and the GoTaq G2 Hot Start Green Master Mix (Promega). Finally, primer pair parameters were determined by quantitative PCR using standards created from the reference sample (Table S5).

All quantitative PCR runs were performed on a StepOnePlus system (Applied Biosystems) with the FastStart SYBR Green Master ROX kit (Roche). A 40 times dilution of each sample cDNA was run in technical triplicate with each primer pair. All samples flagged by the system software for high variability or specificity issues were rerun or removed from the analysis. All run results were analyzed with a relative quantitation method and were normalized first using a triplicate of the D40Ref normalizer sample loaded in all qPCR plates and then with a geometric mean of the results from two reference genes (βactin and 36B4) amplified for all samples. Results are then expressed per condition and timepoint as arithmetic mean of the three biological replicates with SEM as error bars.

### Immunofluorescence on coral samples

Apexes of *S. pistillata* were fixed at 4 °C overnight in 3% paraformaldehyde in S22 buffer (450 mM NaCl, 10 mM KCl, 58 mM MgCl_2_, 10 mM CaCl_2_ 100 mM Hepes, pH 7.8) and then decalcified using 0.5 M ethylenediaminetetraacetic acid (EDTA) in Ca-free S22 buffer at 4 °C. They were dehydrated in an ethanol series and embedded in Paraplast (Leica). 5 µm thick sections were cut from blocks of paraffin-embedded tissues, transfered on glass slides, deparaffinized with 2 × 5 min xylene baths and rehydrated with 5 min baths in decreasing concentrations of ethanol in water (100%, 95%, 70%, 40%, 0%). Antigen retrieval was achieved by incubating slides for 20 min in hot (95 °C) citrate buffer (10 mM citrate, 0.05% Tween 20, pH 6.0) in a steamer and 20 min of cooling at RT. Sections were washed with water and dried overnight. Tissue permeabilization was performed for 1 h at RT in 1× phosphate-buffered saline (PBS); 2 N HCL, 0.5% Triton X-100 followed by two washes with 1× PBS for 5 min. Tissue sections were then fixed in 0.7× PBS, 4% paraformaldehyde, 20 mM sucrose at RT for 15 min with shaking and washed twice with 1× PBS for 5 min. Treatment with 20 µg/ml RNAse was performed in a humid chamber at 37 °C for 1 h in 2× saline–sodium citrate (SSC) buffer and then washed twice with 1× PBS for 5 min. Tissue sections were then incubated for 1 h in saturating buffer (1× PBS, 1% bovine serum albumin [BSA], 0.05% Tween 20, pH 7.4) at 37 °C and rinsed twice with 1× PBS at RT. Samples were incubated overnight at 4 °C in saturating buffer with anti-spFOXO antiserum (0.5 μg/mL) as a primary antibody. After rinsing with saturating buffer, samples were incubated for 2 h with 4 µg/mL Alexa 488-coupled donkey anti-rabbit antibodies (A21206; Molecular Probes) in saturating buffer. After rinsing with PBS (pH 7.4), samples were finally mounted in 4′,6-diamidino-2-phenylindole (DAPI)-containing VectaShield (H-1200; Eurobio scientific) antifade medium. Immunostaining experimental controls were obtained with rabbit pre-immune serum and Alexa 488-coupled anti-rabbit antibodies as described above. Bichannel (DAPI and FITC filter sets) stacks of images were acquired on a DeltaVision elite system (GE Healthcare) epifluorescence microscope with a 60× oil-immersed objective (1.42 NA, Plan Apochromat; Olympus) and a CCD cMOS monochromatic camera. No binning was used and voxel size is 109 µm × 109 µm × 350 µm. All images stacks were then deconvolved and individual in focus optical slices from different conditions were extracted and treated similarly with Fiji software^53^ regarding signal thresholding and amplification to reveal nuclei outlines and antibody specific signal.

### Tissue culture, siRNA transfections and SA-β-galactosidase staining

Human primary MRC-5 cells were obtained from the ATCC, grown at 37 °C, 5% CO_2_ and 5% or 20% O_2_ in DMEM supplemented with 10% fetal calf serum and 100 U/ml penicillin–streptomycin, and regularly tested for mycoplasma contamination.

siRNA On-Target Plus SMARTpools (Dharmacon) targeting specified genes were transfected with Dharmafect1 transfection reagent (Dharmacon) following manufacturer's instructions. For each condition, 150,000 MRC-5 cells in 6-well plates with coverslips were transfected 72 h prior to analyses.

The detection of SA-β‐gal‐positive cells was carried out using the Senescence Detection Kit (Abcam, ab65351) following the manufacturer's instructions.

## Supplementary information

Supplementary Information 1.

Supplementary Information 2.

## Data Availability

Raw transcriptomic data has been deposited on the Gene Expression Omnibus (GEO, https://www.ncbi.nlm.nih.gov/geo/) repository under accession number GSE153706 (https://www.ncbi.nlm.nih.gov/geo/query/acc.cgi?acc=GSE153706).
