# Supplementary material for: Longevity strategies in response to light in the reef coral Stylophora pistillata
Source: Sci Rep. 2020 Nov 17;10:19937. doi: 10.1038/s41598-020-76925-2 (PMC7673115; doi:10.1038/s41598-020-76925-2)
Supplement: Supplementary file 1 — Supplementary Information 1. [file 41598_2020_76925_MOESM1_ESM.pdf]

Supplementary Information

Longevity strategies in response to light in the reef coral *Stylophora pistillata*

Alexandre Ottaviani[1]+*, Rita Eid[1]+, Didier Zoccola[2], Mélanie Pousse[1], Jean-Marc Dubal[1], Edwige Barajas[2], Karine Jamet[1], Kevin Lebrigand[3], Pascal Lapébie[1], Christian Baudoin[1], Marie-Josèphe Giraud-Panis[1], Alice Rouan[1], Gallic Beauchef[4], Christelle Guéré[4], Katell Vié[4], Pascal Barbry[3], Sylvie Tambutté[2], Eric Gilson[1,5]*, Denis Allemand[2]*.

Affiliations

[1] : Medical School of Nice, CNRS, INSERM, IRCAN, Université Côte d'Azur, Nice, France

[2] : Centre Scientifique de Monaco, Monaco, Monaco

[3] : Université Côte d'Azur, CNRS, IPMC, Sophia-Antipolis 06560, France

[4] : Laboratoires Clarins, 12 avenue de la porte des Ternes, 75017 Paris, France

[5] : Department of genetics, CHU, Nice, France

+ These authors contributed equally

* Co-corresponding authors

Supplementary figures legends:

Figure S1. Analysis pipeline. Acknowledgements to Eric Tambutté (CSM) for the *S.pistillata* picture

Figure S2. Bioanalyzer quality controls of total RNA used in sequencing.

Figure S3. Bioanalyzer quality controls of mRNA used in sequencing.

Figure S4. Venn diagram of FOXO targets that are conserved in model organisms and present in the transcriptomic signature. Gene counts per area or species are indicated by bold black numbers. Enrichment P-values for each species are also shown. Genes tested by quantitative polymerase chain reaction (qPCR) are indicated by italics, and those validated are indicated by bold text.

Figure S5. α-spFOXO antiserum detects the endogenous protein. Cropped and inverted image (left) and raw image (right).

Supplementary tables:

Some of these tables being relatively large, they are supplied as corresponding tabs in a single .xlsx file.

Supplementary tables legends:

Table S1. General information on reference gene models and sample reads mapping.

Table S2. Detailed read counts for *Symbiodinium* genes important in photosynthesis.

Table S3. List of annotated differentially expressed coding sequences (CDS).

Table S4. List of transcription factor targets.
a. Published list of target genes for mouse transcription factors.[1–5] **b.** Lists of FOXO target genes in model organisms.[5]

Table S5. List of validated quantitative polymerase chain reaction (qPCR) primers for genes of interest examined in this study.

Supplementary methods:

Coral total RNA extraction

Total RNA for sequencing or qPCR experiments was extracted with Trizol Reagent (Invitrogen) as previously described[6]. Briefly, frozen coral samples were ground into powder in a cryogrinder (Freezer/Mill 6770, Spex Sample Prep) and dissolved in 20 mL Trizol Reagent. Samples were vortexed and incubated at room temperature (RT) for 5 min. Three centrifugations (10 min, 15,000 × *g*, 4°C) were performed to remove the skeleton. We added 4 mL chloroform to the last supernatant, which was vigorously homogenized and incubated for 2–3 min at RT. Centrifugation (15 min, 12,000 × *g*, 4°C) was performed to separate the two phases (aqueous and phenol-chloroform). RNA remained exclusively in the aqueous phase. The aqueous phase was subjected to another chloroform extraction (v/v). We added 10 mL isopropanol to the second aqueous phase, which was vigorously shaken and incubated for 10 min at RT. Centrifugation (15 min, 12,000 × *g*, 4°C) was performed to pellet the RNA. The pellet was resuspended in 5 mL 70% ethanol, vortexed vigorously, centrifuged for 5 min at 7,500 × *g* and 4°C, dried for about 10 min at RT, and then resuspended in 100–200 µL RNase-free water. RNA concentration was determined with a Nanodrop and the Qubit RNA assay kit, more than 2 µg of total RNA was obtained for each sample. In the 48h expression kinetics experiment, all RNA samples quality was assayed with a Bioanalyzer 2100 and RNA 6000 nano kit (5067–1511; Agilent), and 8 out of 117 samples were ruled out for poor quality (RIN inferior to 8, very low quantity, unusual 18S/28S profile, low 260/280 ratio or low 260/230 ratio).

cDNA synthesis

For each quality-checked sample, 2 µg total RNA was treated with DNAse for 15 min at room temperature in the presence of RNAse inhibitors (RNAseOUT; Invitrogen). We stopped reactions by adding EDTA and raising the temperature to 65°C for 5 min. High Capacity RNA-to-cDNA Kit (Applied Biosystems) enzyme and buffer were added before incubation at 37°C for 1 h; we stopped reverse transcription by raising the temperature to 95°C for 5 min.

Reads mapping

Reads mapping and transcriptome index building were performed with Bowtie.[7]

We built the index transcriptome as a colorspace index using the -C Bowtie option from the Bowtie-build command line. We mapped colorspace reads to *S. pistillata* gene models

(available at http://spis.reefgenomics.org/download/Spis.genome.annotation.CDS.longest.fa.gz) and *S. microadriaticum* gene models (available at http://smic.reefgenomics.org/download/Smic.genome.annotation.CDS.longest.sorted.fa.gz) as single-end alignments using the -C Bowtie option.

Differential expression analyses

The number of reads per gene was computed with a Python script (available upon request). Genes with no aligned reads were removed. Differential expression analyses were performed with DESeq2.[8] DEGs were selected with an adjusted P-value cutoff of 0.05. A total of 151 genes in *S. pistillata* were classified as DEGs; fold-change values ranged from 1.6 to 339.

Human orthologs

We retrieved human orthologs using Blastx[9] on the Swiss-Prot database with a cutoff of $E < 1 \times 10^{-3}$. The best hit for each *S. pistillata* gene was selected. We obtained 118 human orthologs from among 151 *S. pistillata* genes.

GO terms and pathway analyses

GO term analyses were performed for the 118 human orthologs with Metascape.[10] Terms with $P < 0.01$, a minimum count of three, and enrichment factor > 1.5 were collected and clustered according to membership similarities. We found 15 GO biological processes, four Kyoto Encyclopedia of Genes and Genomes (KEGG) pathways, and one Reactome pathway database gene set. Pathway analyses of the 118 human orthologs were performed with IPA[11] (QIAGEN Inc., https://www.qiagenbio-informatics.com/products/ingenuity-pathway-analysis) without biased filtering; 103 met our criteria for analyses.

Conversion of UniProtID to mouse genes

Sequences from the UniProtID list were retrieved from the UniProtKB website and used in a Blastp search of the UniProt/Swiss-Prot database for *Mus musculus* (taxid: 10090). For each protein, the best hit with $E < 1 \times 10^{-3}$ was selected for gene symbol and description extraction.

Conversion of gene symbol and description to *Acropora digitifera* genes

The mouse gene symbol was searched on the National Center for Biotechnology Information (NCBI) database for *Acropora digitifera* (taxid: 70779). If the gene symbol was not found, then the gene description was searched.

Production and purification of the FOXO protein

cDNA of the *S. pistillata* FOXO gene (NCBI reference sequence: XM_022933834.1) was cloned into a modified version of pTrcHisB after optimization for *Escherichia coli* expression. spFOXO was produced as a C-terminal his-tag fusion protein for purification. This plasmid was used to transform BL21 *E. coli*-competent cells, which were grown in lysogeny broth (LB) medium containing 100 µg/mL ampicillin at 37°C until an absorbance of 0.7 was reached at an optical density of 600 nm (OD_{600}). Isopropyl β-D-1-thiogalactopyranoside was added for a final concentration of 0.1 mM, and the culture was further incubated at 25°C for 16 h. Cells were harvested by centrifugation at 4,500 × *g* at 4°C for 15 min, and the pellets were used for protein extraction.

This recombinant protein of spFOXO was purified with nickel affinity chromatography (His-Trap HP; GE Healthcare); 900 µg of the protein of interest was collected and sent for antibody production (Covalab).

Rabbit immunization and serum preparation

Six control serum samples were collected from six different rabbits and tested for pre-immune response using a 67-day protocol.

Serum samples were tested by Western blotting using the recombinant protein; three rabbits were selected for protein production. The first inoculation was performed intradermally with 0.5 mL antigen and 0.5 mL Freund's complete adjuvant. The second and the third inoculations were also performed intradermally with 0.5 mL antigen and 0.5 mL Freund's incomplete adjuvant on days 14 and 28. The final immunization was performed subcutaneously with 0.5 mL antigen and 0.5 mL Freund's incomplete adjuvant on day 42. On day 67, final serum samples were collected, supplemented with sodium azide to a final concentration of 0.02%, aliquoted and stored at -80°C.

Supplementary references:

1. Loftus, S. K. *et al.* Hypoxia-induced HIF1α targets in melanocytes reveal a molecular profile associated with poor melanoma prognosis. *Pigment Cell Melanoma Res.* **30**, 339–352 (2017).

2. Han, J. *et al.* ER-stress-induced transcriptional regulation increases protein synthesis leading to cell death. *Nat. Cell Biol.* **15**, 481–490 (2013).
3. Malhotra, D. *et al.* Global mapping of binding sites for Nrf2 identifies novel targets in cell survival response through ChIP-Seq profiling and network analysis. *Nucleic Acids Res.* **38**, 5718–5734 (2010).
4. Acosta-Alvear, D. *et al.* XBP1 controls diverse cell type- and condition-specific transcriptional regulatory networks. *Mol. Cell* **27**, 53–66 (2007).
5. Webb, A. E., Kundaje, A. & Brunet, A. Characterization of the direct targets of FOXO transcription factors throughout evolution. *Aging Cell* **15**, 673–685 (2016).
6. Moya, A. *et al.* Cloning and use of a coral 36B4 gene to study the differential expression of coral genes between light and dark conditions. *Mar. Biotechnol. N. Y. N* **10**, 653–663 (2008).
7. Langmead, B., Trapnell, C., Pop, M. & Salzberg, S. L. Ultrafast and memory-efficient alignment of short DNA sequences to the human genome. *Genome Biol.* **10**, R25 (2009).
8. Love, M. I., Huber, W. & Anders, S. Moderated estimation of fold change and dispersion for RNA-seq data with DESeq2. *Genome Biol.* **15**, 550 (2014).
9. Altschul, S. F., Gish, W., Miller, W., Myers, E. W. & Lipman, D. J. Basic local alignment search tool. *J. Mol. Biol.* **215**, 403–410 (1990).
10. Zhou, Y. *et al.* Metascape provides a biologist-oriented resource for the analysis of systems-level datasets. *Nat. Commun.* **10**, 1523 (2019).
11. Krämer, A., Green, J., Pollard, J. & Tugendreich, S. Causal analysis approaches in Ingenuity Pathway Analysis. *Bioinforma. Oxf. Engl.* **30**, 523–530 (2014).

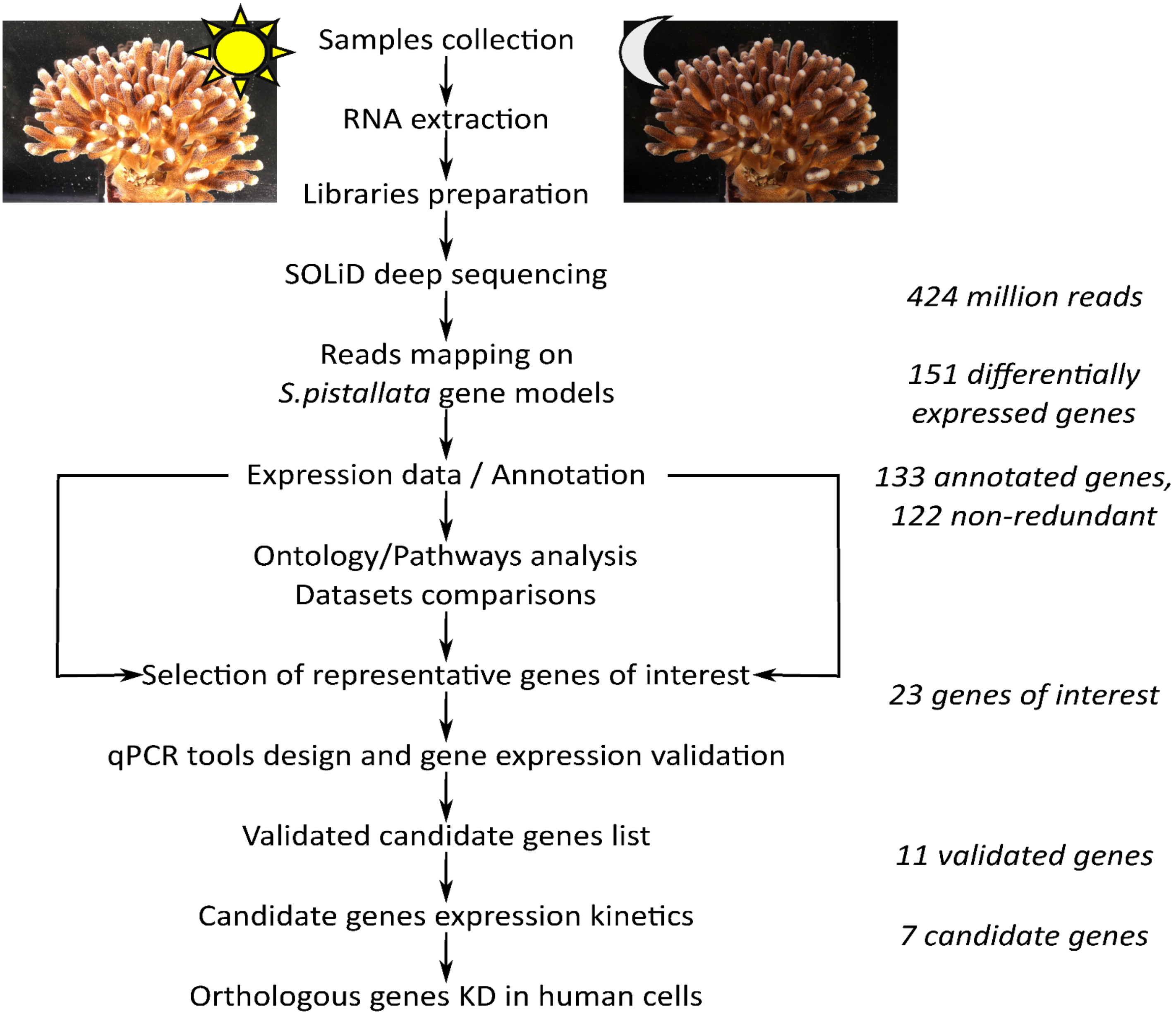

Figure S1. Analysis pipeline

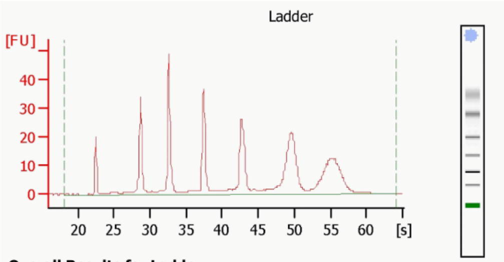

Overall Results for Ladder

RNA Area:	410.3
RNA Concentration:	150 ng/µl
Result Flagging Color:	
Result Flagging Label:	All Other Samples

« Day » samples:

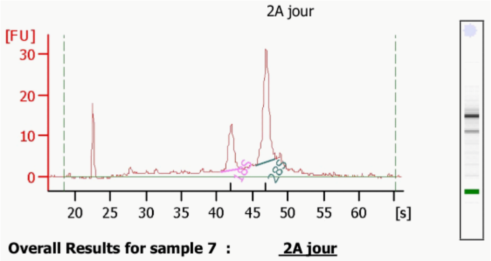

Overall Results for sample 7 : 2A jour

RNA Area:	190.4
RNA Concentration:	70 ng/µl
rRNA Ratio [28s / 18s]:	2.2
RNA Integrity Number (RIN):	8.5 (B.02.08)
Result Flagging Color:	
Result Flagging Label:	RIN: 8.50

Fragment table for sample 7 : 2A jour

Name	Start Time [s]	End Time [s]	Area	% of total Area
18S	40.56	43.20	24.6	12.9
28S	45.52	48.26	55.0	28.9

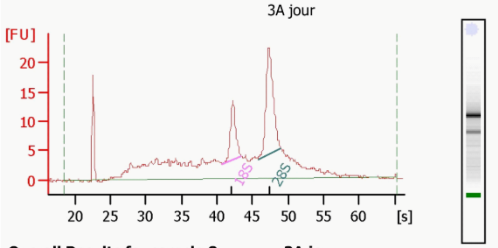

Overall Results for sample 8 : 3A jour

RNA Area:	270.3
RNA Concentration:	99 ng/µl
rRNA Ratio [28s / 18s]:	0.0
RNA Integrity Number (RIN):	7.8 (B.02.08)
Result Flagging Color:	
Result Flagging Label:	RIN: 7.80

Fragment table for sample 8 : 3A jour

Name	Start Time [s]	End Time [s]	Area	% of total Area
18S	40.74	43.42	20.1	7.5
28S	45.81	48.89	44.0	16.3

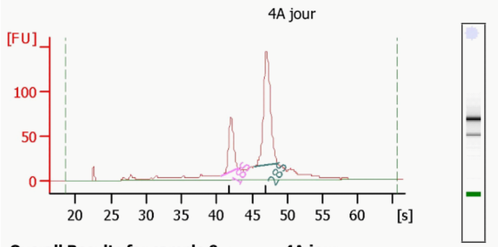

Overall Results for sample 9 : 4A jour

RNA Area:	926.8
RNA Concentration:	339 ng/µl
rRNA Ratio [28s / 18s]:	0.0
RNA Integrity Number (RIN):	9 (B.02.08)
Result Flagging Color:	
Result Flagging Label:	RIN:9

Fragment table for sample 9 : 4A jour

Name	Start Time [s]	End Time [s]	Area	% of total Area
18S	40.58	42.98	115.8	12.5
28S	45.29	48.59	306.2	33.0

« Night » samples:

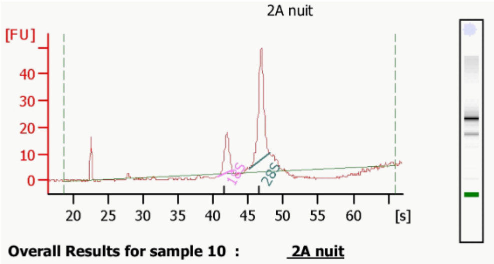

Overall Results for sample 10 : 2A nuit

RNA Area:	158.4
RNA Concentration:	58 ng/µl
rRNA Ratio [28s / 18s]:	0.0
RNA Integrity Number (RIN):	9.2 (B.02.08)
Result Flagging Color:	
Result Flagging Label:	RIN: 9.20

Fragment table for sample 10 : 2A nuit

Name	Start Time [s]	End Time [s]	Area	% of total Area
18S	40.64	42.75	29.7	18.7
28S	45.05	48.06	79.3	50.1

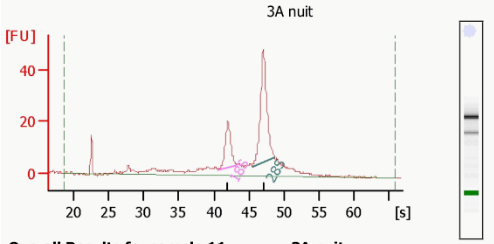

Overall Results for sample 11 : 3A nuit

RNA Area:	331.7
RNA Concentration:	121 ng/µl
rRNA Ratio [28s / 18s]:	0.0
RNA Integrity Number (RIN):	8.6 (B.02.08)
Result Flagging Color:	
Result Flagging Label:	RIN: 8.60

Fragment table for sample 11 : 3A nuit

Name	Start Time [s]	End Time [s]	Area	% of total Area
18S	40.56	43.33	39.9	12.0
28S	45.43	48.63	97.5	29.4

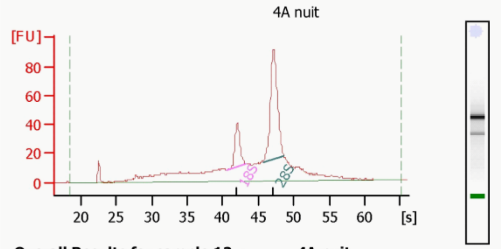

Overall Results for sample 12 : 4A nuit

RNA Area:	796.1
RNA Concentration:	291 ng/µl
rRNA Ratio [28s / 18s]:	0.0
RNA Integrity Number (RIN):	8.1 (B.02.08)
Result Flagging Color:	
Result Flagging Label:	RIN: 8.10

Fragment table for sample 12 : 4A nuit

Name	Start Time [s]	End Time [s]	Area	% of total Area
18S	40.65	43.16	59.2	7.4
28S	45.71	48.62	184.1	23.1

Figure S2. Bioanalyzer quality controls of total RNA used in sequencing

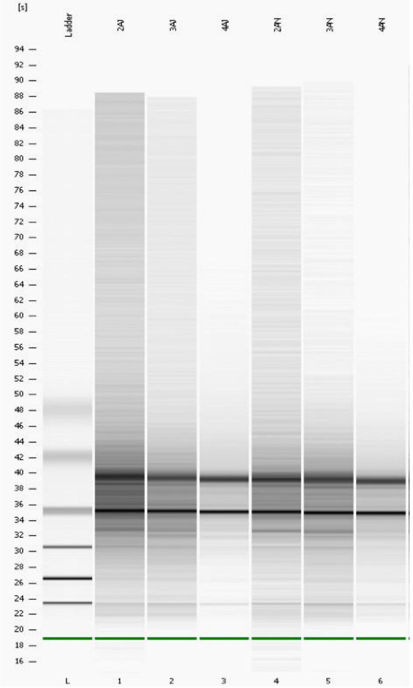

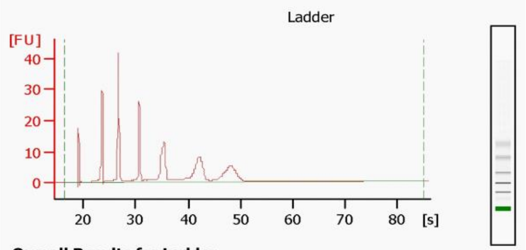

Overall Results for Ladder

RNA Area:	262.3
RNA Concentration:	1,000 pg/µl

« Day » samples:

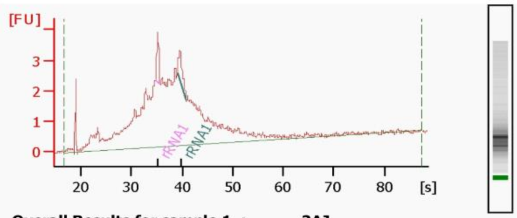

Overall Results for sample 1 : __2AJ__

RNA Area:	99.3
RNA Concentration:	379 pg/µl
rRNA Contamination:	4.4 %

Fragment table for sample 1 : __2AJ__

Name	Start Time [s]	End Time [s]	Area	% of total Area
rRNA1	34.72	35.66	1.9	1.9
rRNA1	39.04	40.92	2.4	2.5

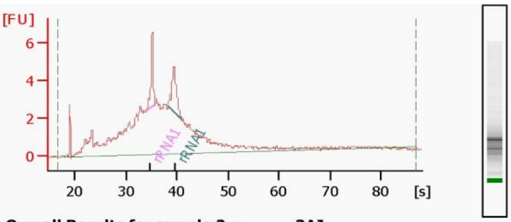

Overall Results for sample 2 : __3AJ__

RNA Area:	135.7
RNA Concentration:	517 pg/µl
rRNA Contamination:	8.9 %

Fragment table for sample 2 : __3AJ__

Name	Start Time [s]	End Time [s]	Area	% of total Area
rRNA1	34.12	35.62	5.4	4.0
rRNA1	38.31	40.90	6.7	4.9

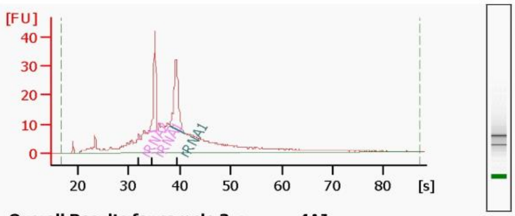

Overall Results for sample 3 : __4AJ__

RNA Area:	516.5
RNA Concentration:	1,969 pg/µl
rRNA Contamination:	21.5 %

Fragment table for sample 3 : __4AJ__

Name	Start Time [s]	End Time [s]	Area	% of total Area
rRNA2	31.72	32.13	1.0	0.2
rRNA1	33.74	35.56	49.5	9.6
rRNA1	38.00	40.96	60.5	11.7

« Night » samples:

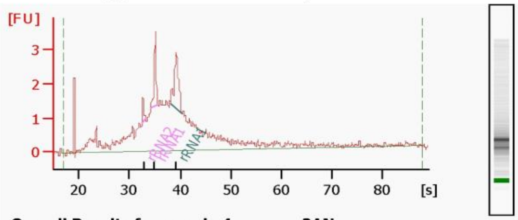

Overall Results for sample 4 : __2AN__

RNA Area:	69.8
RNA Concentration:	266 pg/µl
rRNA Contamination:	11.0 %

Fragment table for sample 4 : __2AN__

Name	Start Time [s]	End Time [s]	Area	% of total Area
rRNA2	32.38	33.17	0.7	1.1
rRNA1	34.12	35.64	2.9	4.1
rRNA1	38.16	40.36	4.1	5.8

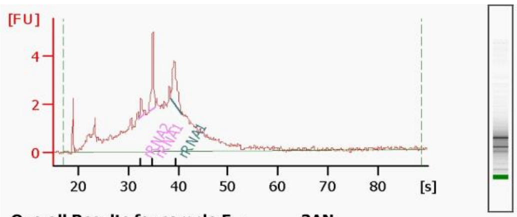

Overall Results for sample 5 : __3AN__

RNA Area:	108.8
RNA Concentration:	415 pg/µl
rRNA Contamination:	9.4 %

Fragment table for sample 5 : __3AN__

Name	Start Time [s]	End Time [s]	Area	% of total Area
rRNA2	32.18	33.03	1.1	1.0
rRNA1	34.03	35.41	4.5	4.1
rRNA1	38.53	40.75	4.7	4.3

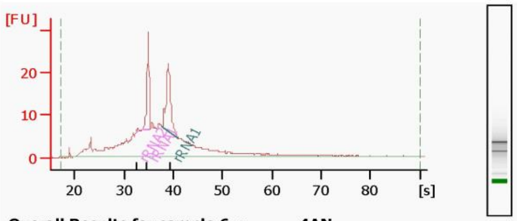

Overall Results for sample 6 : __4AN__

RNA Area:	406.5
RNA Concentration:	1,550 pg/µl
rRNA Contamination:	19.5 %

Fragment table for sample 6 : __4AN__

Name	Start Time [s]	End Time [s]	Area	% of total Area
rRNA2	32.42	33.01	1.1	0.3
rRNA1	33.92	35.42	34.2	8.4
rRNA1	37.79	40.95	44.1	10.8

Figure S3. Bioanalyzer quality controls of mRNA used in sequencing

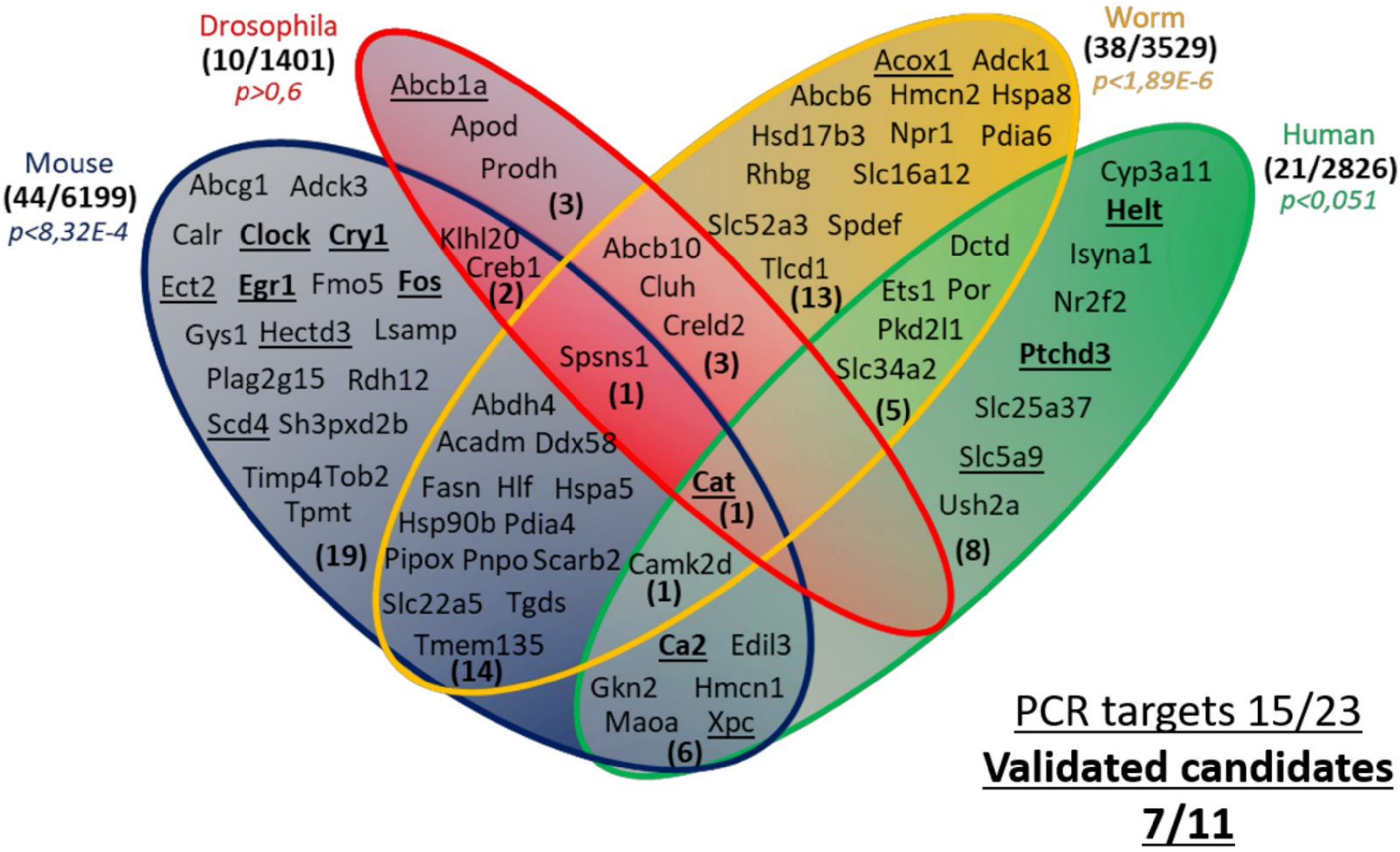

Figure S4. Venn diagram of FOXO targets that are conserved in model organisms and present in the transcriptomic signature

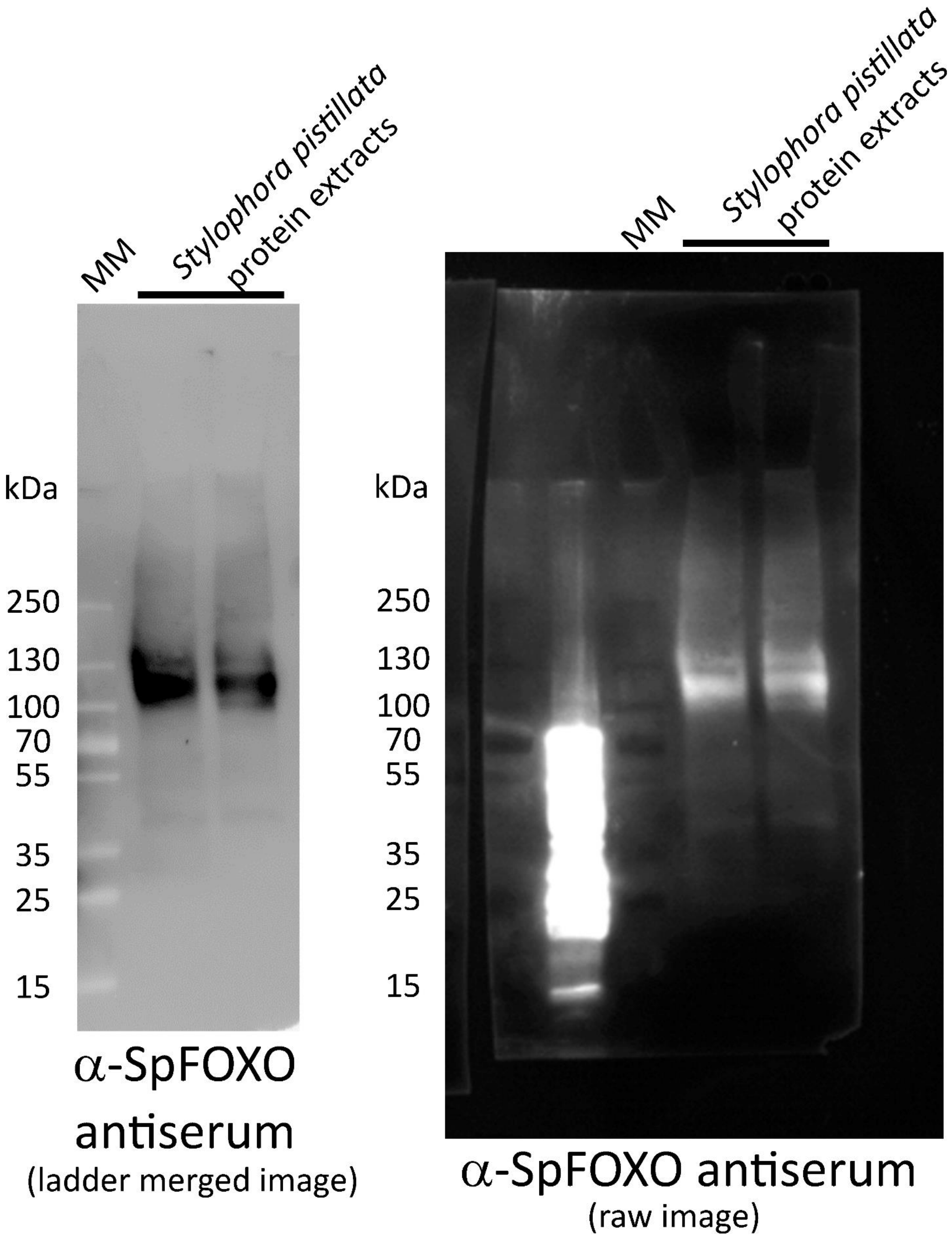

Figure S5. α-spFOXO antiserum detects the endogenous protein
